# Molecular Imaging of Immunity and Inflammation and Its Impact on Precision Medicine

**DOI:** 10.3390/biomedicines9010062

**Published:** 2021-01-11

**Authors:** Moritz Wildgruber, Michel Eisenblätter

**Affiliations:** 1 Department of Radiology, University Hospital, LMU Munich, D-81377 Munich, Germany; 2 Translational Research Imaging Center, Münster University Hospital, D-48149 Münster, Germany; michel.eisenblaetter@uniklinik-freiburg.de; 3 Department of Diagnostic and Interventional Radiology, Freiburg University Hospital, D-79106 Freiburg, Germany

Biomedical research has, over the past decades, found components of the immune system at the functional center of almost every pathophysiological condition and disease. Besides the obvious involvement in inflammatory diseases such as sepsis or autoimmunity, where an overshooting, deregulated immune response is inevitable, players of the innate and adaptive immune system alike were proven to be crucially involved in vascular diseases, rheumatic disorders as well as in multiple steps during cancer development and progression. The role of the immune system components involved can range from aggravation of disease to amelioration, maintaining tissue homeostasis as well as promoting disease resolution. Immunity developed from being a feature of disease development, albeit a crucial one, towards a potential target or means of therapy for a variety of diseases.

The complex role of immune cells in the tumor microenvironment (TME) has become more and more evident and relevant for therapeutic oncology. The complexity mostly lies within the divergent roles of specific immune cells subsets. While some immune cells promote tumor progression and metastasis, e.g., by preparing a premetastatic niche in target organs of subsequent metastasis [1], providing growth factors and promoting neoangiogenesis, other immune cells recognize tumor cells as “foreign”, eliciting an anti-tumor immune response. Enabling and enhancing this anti-tumor immunity has developed into a central pillar of cancer therapy, applying targeted therapies such as checkpoint inhibitors [2]. Although the concept of boosting anti-tumor immunity is biologically convincing, the success of the established therapy regimens is barely predictable, with only a subset of patients responding swiftly and a relevant number of patients not responding at all. Means to predict response to immune-modulating therapy in cancer are largely missing, and tools enabling a selection of patients likely to benefit from personalized, targeted therapy—and moreover to enable an early assessment of therapy response or failure (“fail fast”)—are urgently needed. This is further complicated as tumors exhibit both spatial as well as temporal heterogeneity. Tumor lesions even within the same patient may genetically differ, having originated from different clones of the primary tumor cell, and thus respond differently to highly specific targeted therapy [3]. 

Diagnostics faced a dilemma in this context with a clear lack of measurable markers properly reflecting the complexity of tumor-immune interaction: Common systemic diagnostic approaches such as liquid biopsy or other serum tumor markers fail to represent the interlesional heterogeneity. Similarly, obtaining biopsies of all different tumor lesions, reflective of inter- and intralesional heterogeneity, is practically not feasible. Only imaging has a potential to provide a comprehensive image of the tumor, the tumor microenvironment and the systemic heterogeneity. 

Clinical imaging as applied today unfortunately lacks both sensitivity and specificity to address dedicated aspects of (anti-) tumor immunity. Traditional assessment of anticancer therapy by using Response Evaluation Criteria in Solid Tumors (RECIST) does not reflect the effects of novel targeted therapies and has shown to fail in the differentiation of responders and non-responders or is succeeding only too late after initiation of treatment [4]. Thus, traditional cross-sectional imaging techniques such as computed tomography and magnetic resonance imaging have major deficits in assessing molecular oncology. By extracting and mathematically analyzing information hidden within morphological images, radiomics is explored as one approach to get more relevant biomarkers from basically standard examinations. Equivalent approaches involving dedicated mathematics and the use of artificial intelligence (AI) are being explored for genetic information (genomics) and protein expression patterns (proteomics). However, even the emerging “-omics” approaches offer only snapshots of dedicated biomarkers. Imaging can take this information to another level, following the activity of these markers in space and over time [5]. 

Oncology is just one example of molecular imaging as an expected driver of personalized medicine, triggered via molecular diagnostics. 

Equivalent demand and equivalent challenges can be seen in almost all other fields of clinical medicine. In cardiology, the prediction of cardiovascular events is one of the major challenges. Similar to oncology, conventional biomedical imaging has failed to predict high-risk atherosclerotic lesions, prone to rupture, subsequently causing clinical events such as myocardial infarction or stroke. It is possible to identify and visualize key processes of vascular inflammation and vulnerable plaques, such as high lesional macrophage content and high concentrations of proteases that destabilize the plaque towards rupture [6], using molecular imaging. The major advantage of imaging over other molecular diagnostics is that it provides a more holistic picture of inflammation and immune activation, covering the entire organism. In this context, the activation of the hematopoietic system, such as the spleen and the bone marrow in patients with atherosclerosis and cardiovascular disease, can be depicted by 18F Fluorodeoxyglucose (FDG) PET imaging [7,8].

A major hurdle for translation of -omics approaches—especially those combining information from imaging, genetics and proteome—is the purely statistical nature of the results. A pathological relevance may be evident, but a biological representation or correlation is hard to define. Still, integration of diagnostic information might change the clinical course of imaging. 

At the same time, highly specific imaging of molecular and cellular processes remains in high demand today from a clinical perspective—for example, as a screening tool for targeted therapy—as well as in basic research. 

Various molecular imaging strategies have been developed over the last decade. By labeling specific antibodies with signaling molecules, the expression of major tumor antigens can be visualized. Thereby, central hallmarks of cancer can be addressed, such as tumor-promoting inflammation, invasion and metastasis, angiogenesis and resistance towards cell death. Various techniques addressing metabolic features, such as positron emission tomography and hyperpolarized and CEST (Chemical Exchange Saturation Transfer) MRI, have greatly added to understanding how targeted therapies do influence tumor metabolism.

While many of these novel molecular imaging approaches have proven to work well in the preclinical setting, the translation of these techniques into clinical practice is still limited. While optimized animal models provide an ideal background for specific imaging approaches, diagnostic performance in the patient would mean balancing specificity and the limited sensitivity of clinical imaging modalities. Additionally, pharmacodynamic differences between animal models and humans as well as toxicity issues further limit clinical translation, with all challenges cemented in regulatory hurdles set for diagnostic agents before regular clinical application.

The assessment of immune cell dynamics used to be the domain of experimental, invasive techniques such as intravital microscopy, which disrupts the integrity of the organism and at the same time provides only a small spatial window to the immune system. Novel techniques such as time-lapse MRI enable non-invasive tracking of labelled immune cells in various states of local and systemic inflammation in vivo over time [9,10]. Ex vivo imaging techniques such as mass spectrometry imaging allow for quantification of imaging substrates with high spatial resolution, enabling and supporting novel -omic approaches [9,11,12].

How to cross the translational gap between promising preclinical research and clinical routine remains the big question in imaging research.

Radionuclide imaging may have the greatest potential. In contrast to optical imaging techniques, also including optoacoustic imaging, nuclear imaging techniques do not suffer from rapidly decreasing signal-to-noise in deeper tissue regions but show almost unlimited tissue penetration. Moreover, tracer production according to good manufacturing practices (GMP) conditions is established as routine in many nuclear medicine laboratories. A radioactive ligand targeted towards matrix metalloproteinases has recently shown to effectively visualize and quantify the inflammatory burden in patients with multiple sclerosis [13].

In summary, molecular imaging in the preclinical setting allows for tracking of specific players of the innate and adaptive immune system in vivo. The non-invasive nature of most approaches allows for repetitive monitoring of inflammation and immunity which enables one to identify specific disease targets, define the optimal time points for therapeutic intervention and allow for early monitoring of therapies modulating the immune response. This special issue aims to collect some fine examples of molecular imaging both in the preclinical field but also their translation towards clinical precision medicine.
